# The mating type locus protein MAT1-2-1 of *Trichoderma reesei* interacts with Xyr1 and regulates cellulase gene expression in response to light

**DOI:** 10.1038/s41598-017-17439-2

**Published:** 2017-12-11

**Authors:** Fanglin Zheng, Yanli Cao, Lei Wang, Xinxing Lv, Xiangfeng Meng, Weixin Zhang, Guanjun Chen, Weifeng Liu

**Affiliations:** State Key Laboratory of Microbial Technology, School of Life Science, Shandong University, No. 27 Shanda South Road, Jinan, 250100 Shandong P. R. China

## Abstract

Cellulase production in the model cellulolytic fungus *Trichoderma reesei* is subject to a variety of environmental and physiological conditions involving an intricate regulatory network with multiple transcription factors. Here, we identified the mating type locus protein MAT1-2-1 as an interacting partner for the key transcriptional activator Xyr1 of *T. reesei* cellulase genes. Yeast two-hybrid and GST pulldown analyses revealed that MAT1-2-1 directly interacted with the putative transcription activation domain (AD, 767~940 aa) and the middle homology region (MHR2, 314~632 aa) of Xyr1. Disruption of the *mat1-2-1* gene compromised the induced expression of cellulase genes with Avicel in response to light or with lactose. Chromatin immunoprecipitation (ChIP) demonstrated that MAT1-2-1 was recruited to the *cbh1* (cellobiohydrolase 1-encoding) gene promoter in a Xyr1-dependent manner. These results strongly support an important role of MAT1-2-1 as a physiological cofactor of Xyr1, and suggest that MAT1-2-1 represents another regulatory node that integrates the light response with carbon source signaling to fine tune cellulase gene transcription.

## Introduction

The ascomycete *Trichoderma reesei* (teleomorph *Hypocrea jecorna*), a saprophytic filamentous fungus, represents an important workhorse for industrial production of cellulases as well as other proteins. The secreted cellulase mixture synergistically decomposes the insoluble cellulosic materials to fermentable sugars^1^. In *T. reesei*, most of the cellulase genes are coordinately controlled by a suite of transcription factors^2^. Among others, xylanase regulator 1 (Xyr1) is absolutely necessary for activating the expression of most cellulases/hemicellulases. Lack of Xyr1 has been shown to eliminate cellulase induction by almost all inducers (such as cellulose and lactose)^3^. Regulation of Xyr1 expression and/or activity has thus been considered to be crucial for the production of various hydrolytic enzymes in *T. reesei* though the exact mechanism remains largely elusive^4–6^. Apart from the extensively studied carbon source-dependent expression of cellulase genes, other environmental cues, especially light, have also been found to exert a profound effect on cellulase production^7–11^. Our understanding on cellulase gene regulation in this filamentous fungi is thus far from complete.

Recently, *T. reesei* has been demonstrated to be able to carry out a heterothallic sexual cycle and successful mating has thus been achieved in this industrial fungus^12,13^. Similar to other filamentous ascomycete fungi, *T. reesei* has two mating types, *MAT1-1* and *MAT1-2*, with *MAT-*idiomorphs occupying the same genomic region in the respective types. Whereas the *MAT1-1* locus contains three genes, the *MAT1-2* locus encodes a single protein with a high motility group (HMG) domain^12^. Moreover, the composition of the pheromone system in *T. reesei* is almost the same as other ascomycete fungi except that a novel h-type pheromone peptide (Hpp1) with characteristics of a- and α-type exists instead of the classical a-type peptide pheromone precursor^14^. Unlike other fungi, it has been found that neither pheromone precursor genes nor pheromone receptor genes of *T. reesei* are transcribed in a strictly mating type dependent manner although enhanced expression levels have been observed in the cognate mating type^15^. Another peculiarity in *T. reesei* is that sexual development is more efficient in light and upon growth on rich medium^12^. Potential signaling pathways transmitting these light signals in controlling development have been extensively investigated^11,16,17^. In particular, both the PAS domain protein ENVOY (ENV1) and the light-dependent regulator VEL1 have been found to be important for balanced regulation of pheromone precursor genes and receptor genes largely in a mating type dependent manner, and thus crucial for successful mating^16,17^. On the other hand, both regulators have been also found to be involved in regulation of cellulase gene expression^7,11^.

In this study, we screened for proteins interacting with Xyr1 using the yeast two-hybrid system and identified the mating type locus protein MAT1-2-1 as an interacting partner. We further provided insights into the role of MAT1-2-1 in regulation of cellulase gene expression in light. MAT1-2-1 may thus represent another regulatory node that integrates the light response with carbon source signaling to fine tune cellulase gene transcription.

## Results

### Mat1-2-1 interacts with Xyr1 in a yeast two-hybrid screen

To explore the mechanism of Xyr1 mediating the transcriptional activation of cellulase genes in *T. reesei*, a yeast two-hybrid (Y2H) screen was performed to isolate any protein that would interact with Xyr1^18^. With the C-terminal putative activation domain (AD) of Xyr1 (767~940 aa) as a bait, a *T. reesei* cDNA expression library prepared under cellulase-inducing conditions was transformed into the reporter strain and transformants were selected on the quadruple dropout medium (QDO, SD/–Ade/–His/–Leu/–Trp) containing 100 ng/ml AbA. A positive clone with a 0.73 Kb cDNA insert was repeatedly obtained in three independent screens. Sequencing analysis of the cDNA insert revealed a full-length open reading frame of 241 amino acids corresponding to the mating type locus (*MAT1-2*) encoding protein, also annotated as a mating related transcription factor MAT1-2-1 (Tr_124341).

**Figure 1:**
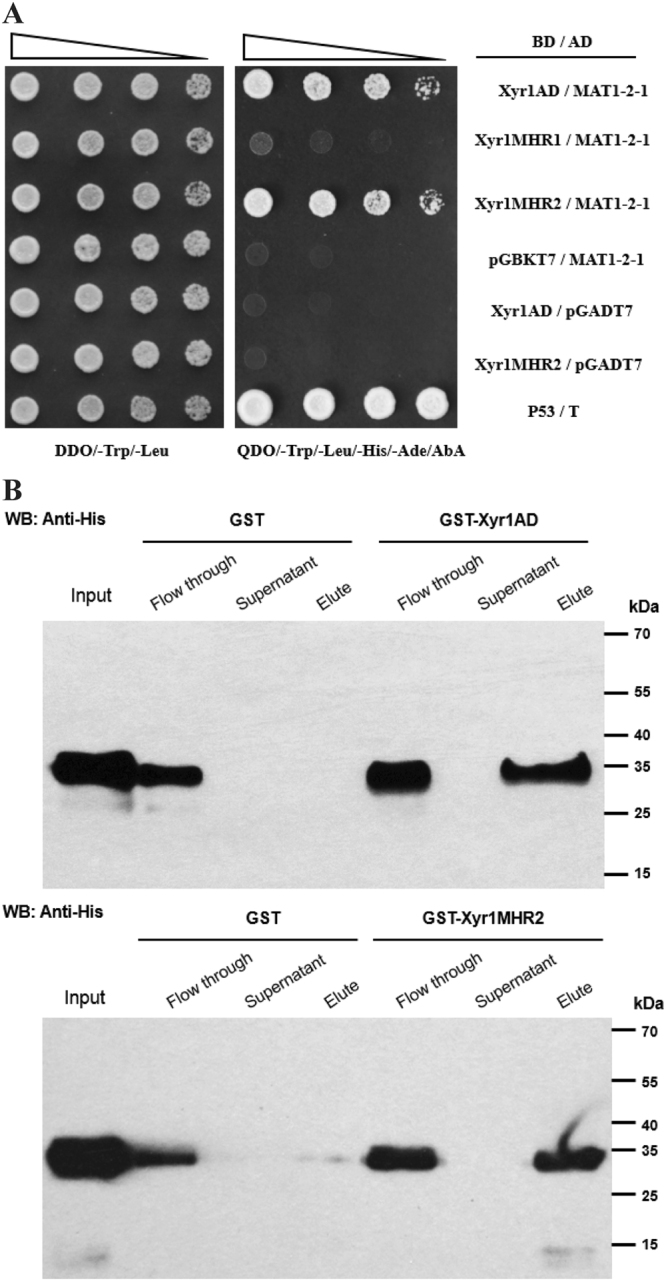
MAT1-2-1 interacts with Xyr1 *in vitro*. (**A**) Yeast two-hybrid analyses of the interaction between MAT1-2-1 and different regions of Xyr1. Serial dilutions of yeast transformant cells harboring the indicated plasmids were spotted on double dropout medium (DDO, SD/–Leu/–Trp) and quadruple dropout medium (QDO, SD/–Ade/–His/–Leu/–Trp) containing AbA plates, respectively, and were allowed grow at 30 °C for 3 days. Transformant containing p53/T pair was used as the positive control. (**B**) MAT1-2-1 interacts with Xyr1 AD and Xyr1 MHR2 in an *in vitro* GST pull-down assay. Recombinant 6 × His-MAT1-2-1 was incubated with glutathione sepharose 4B bead-coupled GST-Xyr1 AD, GST-Xyr1 MHR2 or GST as a control. MAT1-2-1 distributed in different fractions of the assay was detected by western blot with anti-His antibody.

To see whether other regions of Xyr1 were also involved in the interaction with MAT1-2-1, the same Y2H assay was performed with different regions of Xyr1 as the bait. As with AD, a middle homology region of Xyr1 (MHR2, 314~632 aa) was also found to interact with MAT1-2-1 (Fig. 1A). However, an extended MHR region (MHR1, 314~772 aa) failed to support the activation of reporter genes (Fig. 1A). To further verify the interaction, we performed a pull-down assay. In accordance with the results of Y2H, His-tagged MAT1-2-1 was efficiently retained by recombinant GST-Xyr1AD and -Xyr1MHR2, whereas none of them could be detected in the GST only coupled beads (Fig. 1B). Taken together, these results indicate that MAT1-2-1 directly interacts with different regions of Xyr1 *in vitro*.

### MAT1-2-1 is a nuclear protein that co-localizes with Xyr1 and its transcription is upregulated by light or on inducing carbon sources

**Figure 2:**
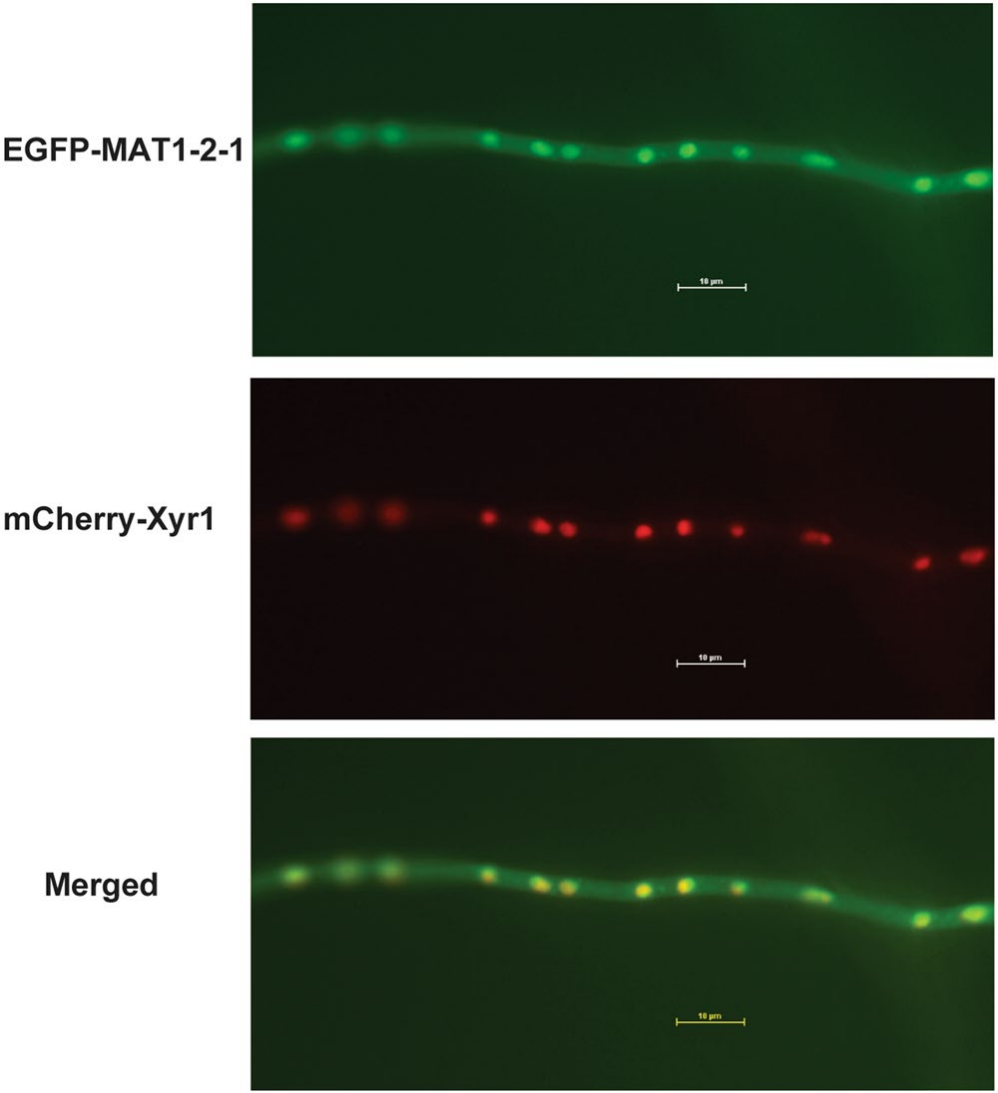
MAT1-2-1 is co-localized with Xyr1 in the nucleus. Germinated hypha of *T. reesei* transformant simultaneously expressing EGFP-MAT1-2-1 and mCherry-Xyr1 were analyzed for the respective fluorescence with a Nikon Eclipse 80i fluorescence microscope. The result shown represents one of at least two independent experiments.

To investigate the subcellular localization of MAT1-2-1, a recombinant strain that simultaneously expressed EGFP-MAT1-2-1 and mCherry-Xyr1 both under the control of a copper responsive promoter P*tcu1* was constructed^19^. As shown in Fig. 2, EGFP-MAT1-2-1 fluorescence was readily observed to be distributed as a series of dots across the hypha and matched well with mCherry-Xyr1 fluorescence, which has been reported to be rapidly imported into the nucleus upon induced expression^6^. MAT1-2-1 also remained in the nucleus when transferred to medium containing inducing carbon sources including Avicel or lactose (data not shown). These results thus indicate that MAT1-2-1 is mainly a nuclear protein that co-localizes with Xyr1 regardless of the carbon source.

**Figure 3:**
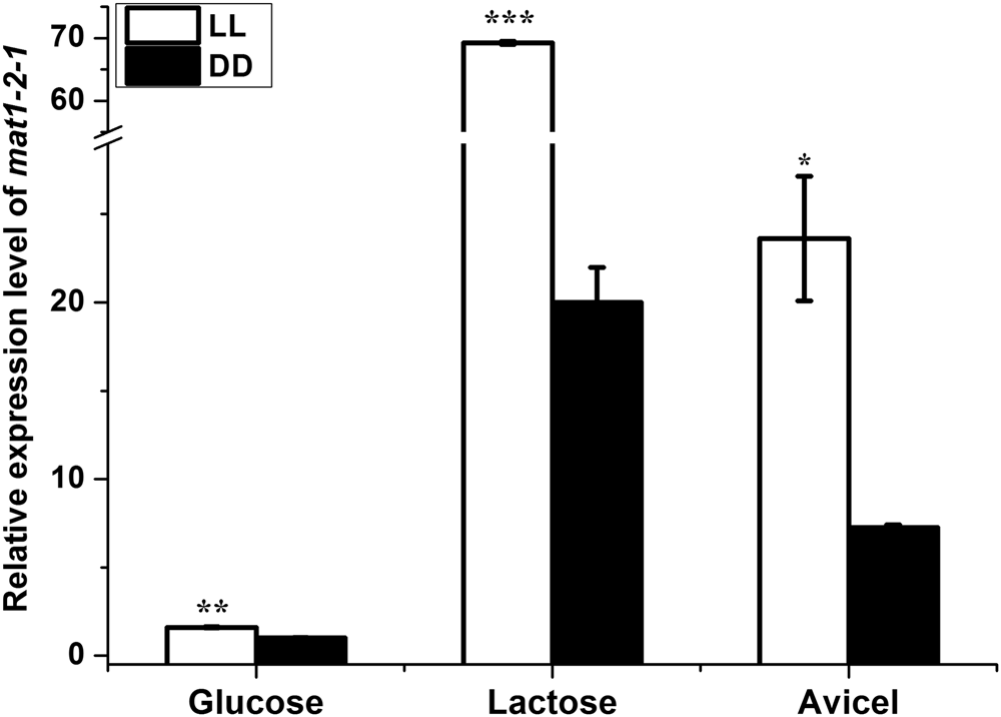
Transcription of *mat1-2-1* under different cultivation conditions. qRT-PCR analysis of the relative transcription level of *mat1-2-1*. The mean values of each column were obtained from three independent biological replicates. Error bars indicated the standard error (SE) from three independent biological replicates. Significant differences (T-test *P < 0.05, **P < 0.01, ***P < 0.001) were detected for the transcription of *mat1-2-1* when comparing light and darkness for the indicated inducing carbon sources.

The transcription of *mat1-2-1* under different cultivation conditions was determined by RT-qPCR (Fig. 3). Whereas the transcription of *mat1-2-1* was up-regulated on inducing carbon sources compared to that on repressing glucose, the upregulation was found to be more dramatic on lactose than on Avicel. In accordance with previous report^20^, the enhanced expression of *mat1-2-1* on both carbon sources was further boosted under constant light (LL) compared to that on constant darkness (DD). These results implied that MAT1-2-1 may play a role in induced cellulase synthesis in response to nutrient and light signals.

### MAT1-2-1 modulates the induced cellulase gene expression in response to light

**Figure 4:**
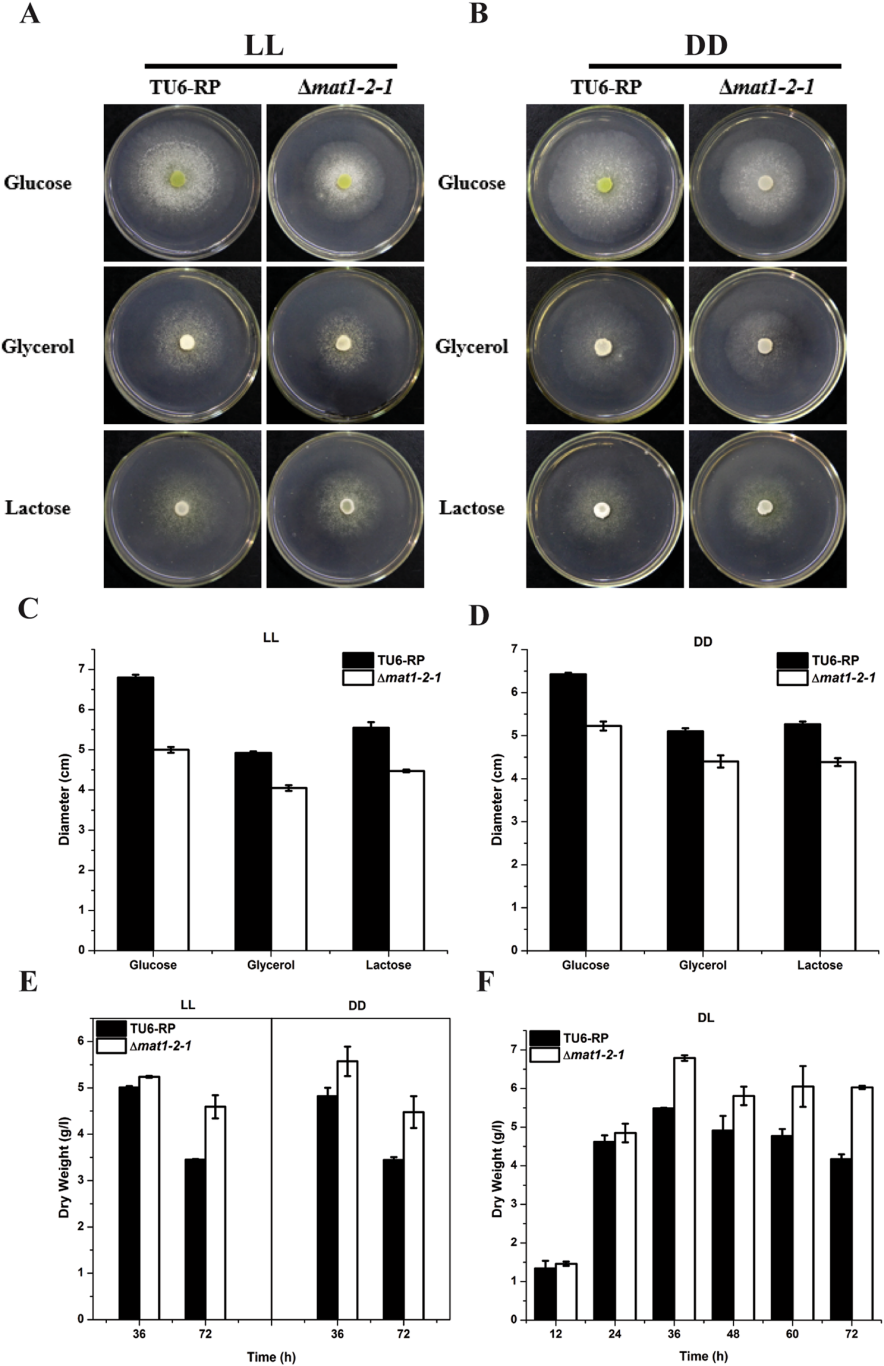
Growth assay of the TU6-RP and Δ*mat1-2-1* strains on solid agar plate or in liquid MA medium under constant light (LL) or darkness (DD) condition. (**A** and **B**) Growth of the Δ*mat1-2-1* and TU6-RP strains on MM agar plates with the indicated carbon sources under constant light and darkness conditions, respectively. (**C** and **D**) The diameter of the colony measured after three days of growth. (**E** and **F**) Dry weight of the Δ*mat1-2-1* and TU6-RP strains grown in liquid MA medium with 1% glucose (w/v) at the indicated time points in constant light/darkness or in daylight with normal light-dark cycle.

**Figure 5:**
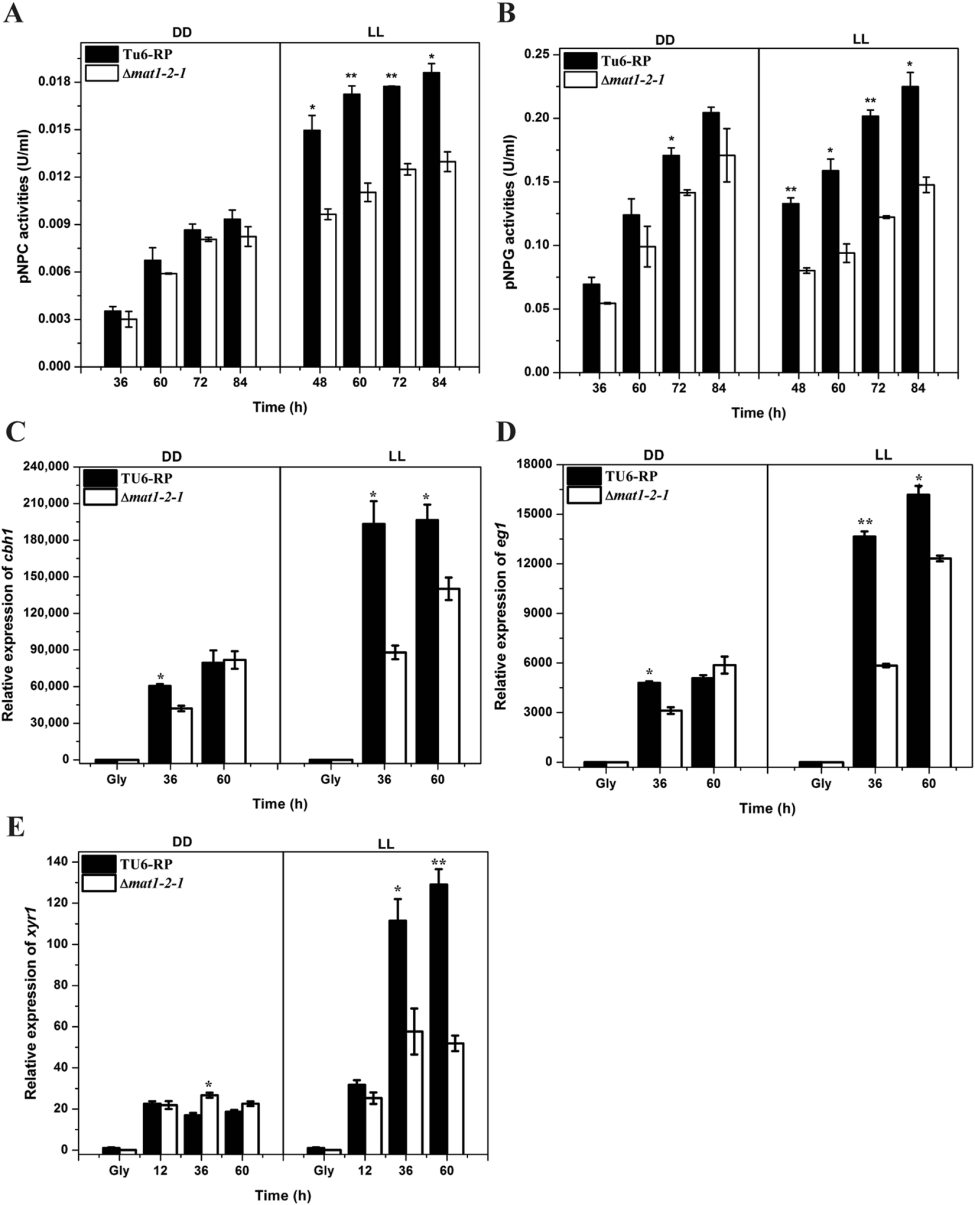
Deletion of *mat1-2-1* leads to down-regulation of cellulase gene expression on cellulose in light but not in darkness. (**A** and **B**) *p*NPC and *p*NPG hydrolytic activities of the extracellular supernatant of the Δ*mat1-2-1* and TU6-RP strains. (**C** to **E**) Relative expression levels of *cbh1* (**C**), *eg1* (**D**) and *xyr1* (**E**) in these two strains cultured under the same conditions for enzymatic activity analyses were assayed by RT-qPCR. Error bars indicated the standard error (SE) from three independent biological replicates. Significant differences (T-test *P < 0.05, **P < 0.01) were detected for the extracellular activities and the transcription of the *cbh1*, *egl1* and *xyr1* genes between TU6-RP and the Δ*mat1-2-1* strain for the indicated time points after induction.

Considering the detected interaction between MAT1-2-1 and Xyr1, the role of MAT1-2-1 in cellulase gene expression was investigated. A *T. reesei* mutant lacking the *mat1-2-1* gene was obtained by targeted gene replacement with the orotidine-5-decarboxylase gene *pyr4* in TU-6 strain. PCR verification and Southern blot analysis confirmed that the deletion of *mat1-2-1* occurred as predicted, integrating within the *T. reesei* genome resulting in the removal of the expected coding sequences (Fig. S1). Whereas deletion of *mat1-2-1* resulted in a slightly slower growth rate on agar plate (Fig. 4A–D), the final biomass of the mutant strain cultured in liquid MA medium with glucose as the sole carbon source was slightly higher than that of the parental strain (Fig. 4E and F). Considering the light-responsive transcription of *mat1-2-1*, the induced production of cellulase activity in the mutant and the parental strains was analyzed on Avicel in either constant light or darkness by measuring the extracellular *p*NPC and *p*NPG hydrolytic activities. Consistent with previous report^7^, the induction of cellulases was stimulated by light compared to that in constant darkness (Fig. 5A and B). The results also revealed that the extracellular *p*NPC and *p*NPG hydrolytic activities were significantly decreased in the Δ*mat1-2-1* mutant compared to those in the parental strain under constant light condition, whereas hardly any effect was observed in darkness except that only a slight decrease in *p*NPG hydrolytic activities was observed at 72 h of induction. A similar light-dependent effect of the *mat1-2-1* deletion on the induced xylanase activities was also observed in media using Avicel or xylan as the sole carbon source (Fig. S2). Further examination of the endogenous *cbh1* and *eg1* mRNA by RT-qPCR demonstrated that the decreased cellulase activities as observed in the *mat1-2-1* deletion strain resulted from a down-regulation in the steady state transcripts of cellulase genes (Fig. 5C and D). Similarly, the transcription of the key transcriptional activator gene *xyr1* was significantly decreased on Avicel in the absence of MAT1-2-1 in light but not in dark compared with that of the control strain (Fig. 5E).

### MAT1-2-1 plays an important role in lactose induced expression of cellulases

**Figure 6:**
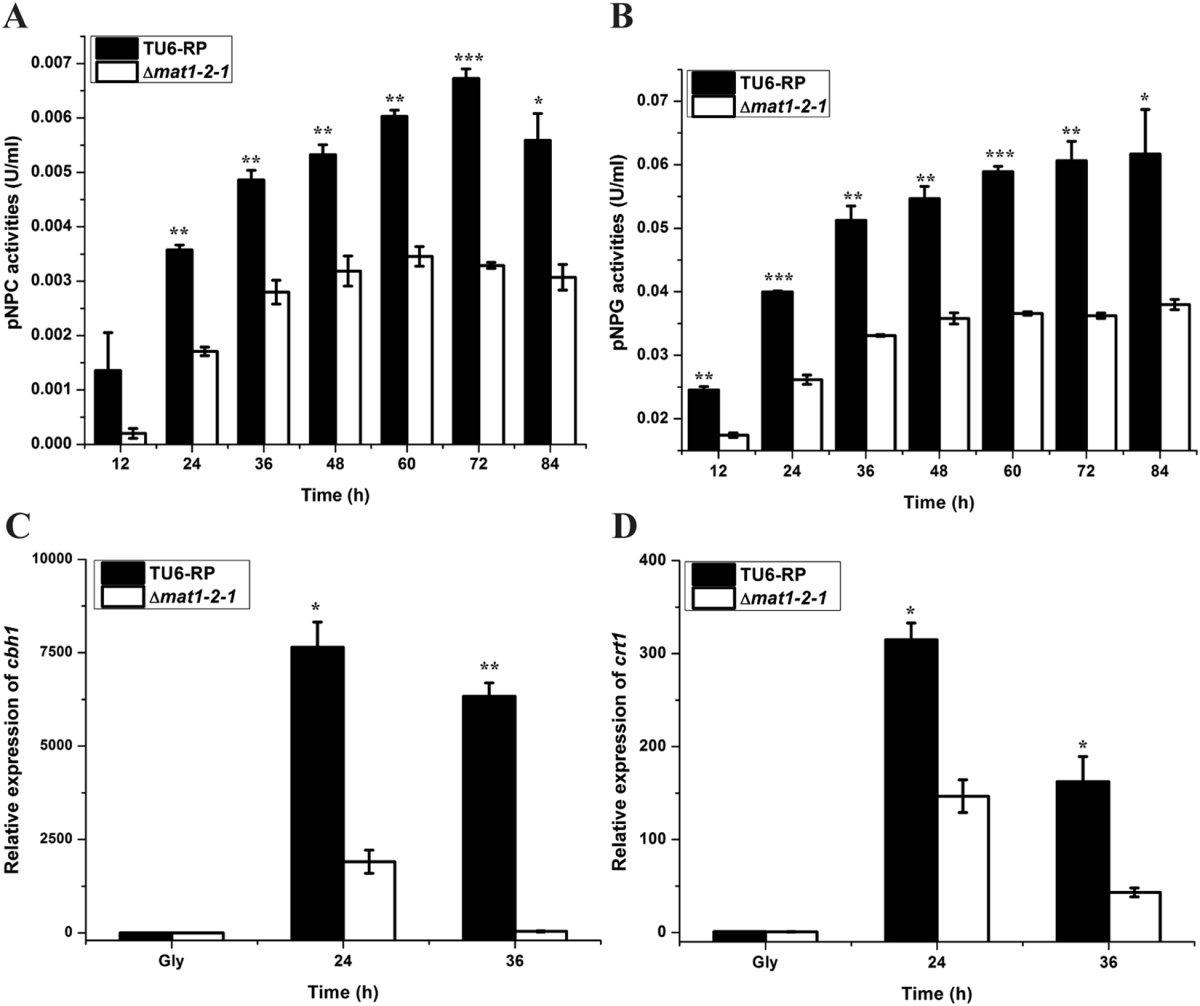
MAT1-2-1 plays an important role in lactose induced expression of cellulases. (**A** and **B**) *p*NPC and *p*NPG hydrolytic activities of the extracellular supernatant, and (**C** and **D**) the relative transcription levels of *cbh1* and *crt1* of the Δ*mat1-2-1* and TU-6-Rp strains cultured on lactose. Error bars indicated the SE from three independent biological replicates. Significant differences (T-test, *P < 0.05, **P < 0.01, ***P < 0.001) were detected for the extracellular *p*NPC and *p*NPG activities between TU6-RP and Δ*mat1-2-1* for the indicated time points after induction. Significant differences also detected for the transcription of the *cbh1* and *crt1* genes (T-test *P < 0.05, **P < 0.01).

In contrast to the case on Avicel, light does not stimulate *T. reesei* cellulase gene expression on lactose but rather has a slightly negative effect^21^. Considering that transcription of *mat1-2-1* also showed a significant upregulation on lactose even under constant darkness (Fig. 3), the effect of the absence of MAT1-2-1 on lactose induced cellulase expression was investigated. Cultivation of the Δ*mat1-2-1* and TU6-RP strains was performed under normal daylight condition (light–dark cycles) with 1% lactose (w/v) as the sole carbon source, wherein the induced cellulase synthesis in TU6-RP strain was comparable to that in darkness (data not shown). Analyses of the extracellular *p*NPC and *p*NPG hydrolytic activities revealed that deletion of *mat1-2-1* resulted in a significantly decreased level of extracellular cellobiohydrolase and β-glucosidase activities (Fig. 6A and B). Transcriptional analyses by RT-qPCR further revealed that the expression level of the major cellulase gene *cbh1* was dramatically decreased in the Δ*mat1-2-1* strain than that of the TU6-RP (Fig. 6C). Deletion of *mat1-2-1* also resulted in a significant down-regulation of the MFS superfamily transporter *crt1* (Fig. 6D), which has been reported to be essential for lactose uptake and cellulase induction by lactose^22,23^. Taken together, these results indicate that MAT1-2-1 also plays an important regulatory role in celllulase gene expression on lactose.

### MAT1-2-1 is recruited to the *cbh1* promoter in a Xyr1-dependent manner

**Figure 7:**
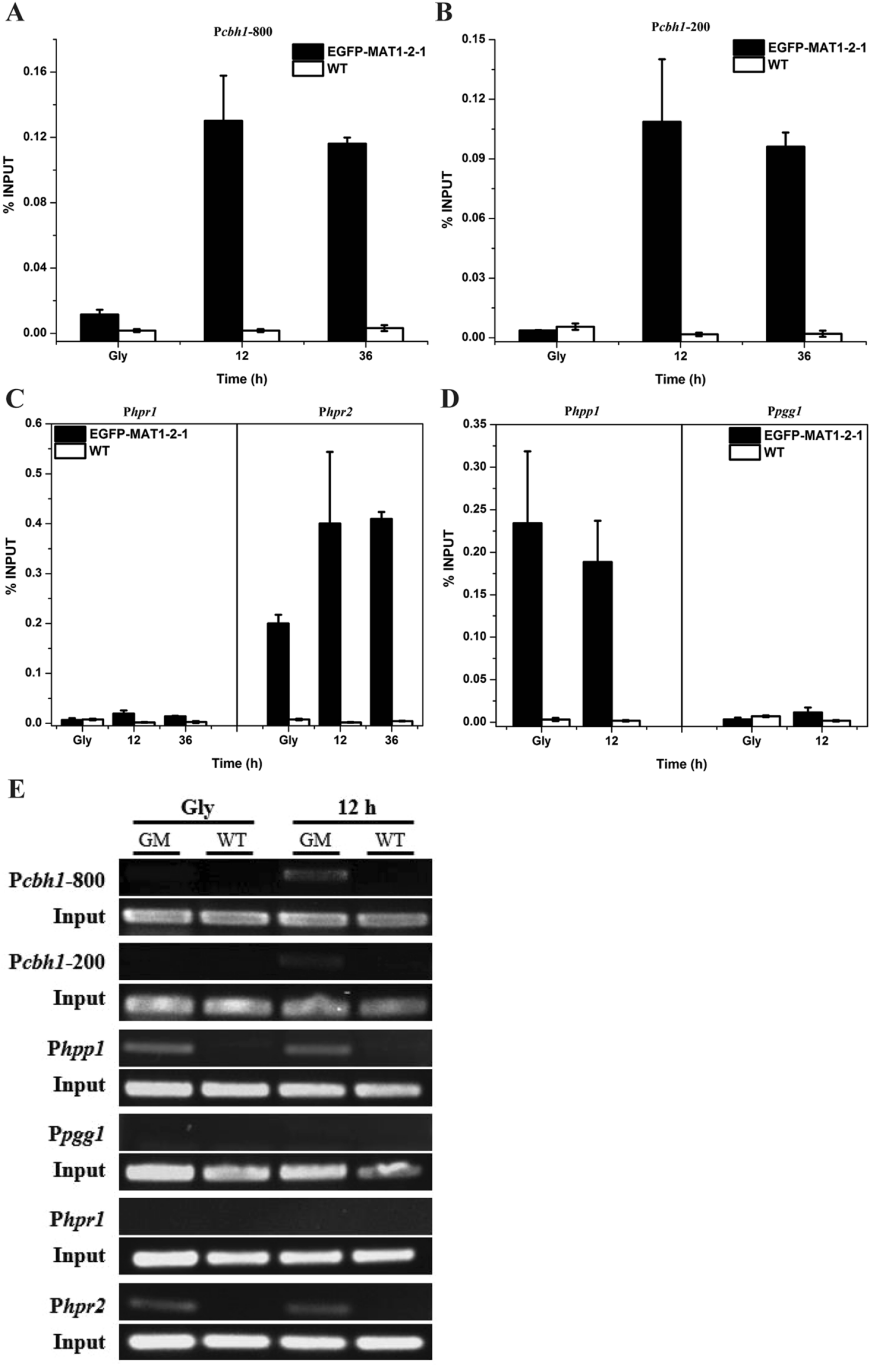
MAT1-2-1 is recruited to the *cbh1* promoter upon cellulase induction. ChIP analyses of MAT1-2-1 recruitment to the −800 and −200 regions of the *cbh1* promoter (**A** and **B**), the pheromone receptor gene promoters (*hpr1* and *hpr2*) (**C**), and the pheromone precursor gene promoters (*hpp1* and *pgg1*) (**D**) before (Gly) and after induction with Avicel for the indicated time periods. (**E**) Semi-quantitative PCR products amplified from the above promoter fragments in the immunoprecipitated samples. GM and WT denoted samples from the EGFP-MAT1-2-1 and TU6-RP strains, respectively. Gly denoted pre-cultures on glycerol.

To test the possibility that MAT1-2-1 is recruited to cellulase gene promoters and thus is directly involved in regulation of cellulase gene expression upon induction, chromatin immunoprecipitation (ChIP) experiments were performed using the GFP antibody followed by quantitative real-time PCR in a *T. reesei* recombinant strain expressing EGFP-MAT1-2-1. The results demonstrated that, in contrast to the non-inducing conditions with glycerol as the sole carbon source, MAT1-2-1 was efficiently recruited to the −800 and −200 bp regions of the *cbh1* promoter under inducing conditions (Fig. 7A,B and E). The same two regions were also found to be more efficiently occupied by Xyr1 whose expression only occurred upon induction^24^. As a control, relatively higher MAT1-2-1 occupancy was found on the promoters of the pheromone gene *hpp1* and its cognate receptor gene *hpr2* regardless of the culture conditions, whereas no such enrichment occurred on promoters of *pgg1* and *hpr1*, thus indicating that MAT1-2-1 was also directly involved in the transcriptional regulation of *hpp1* and *hpr2* (Fig. 7C–E).

**Figure 8:**
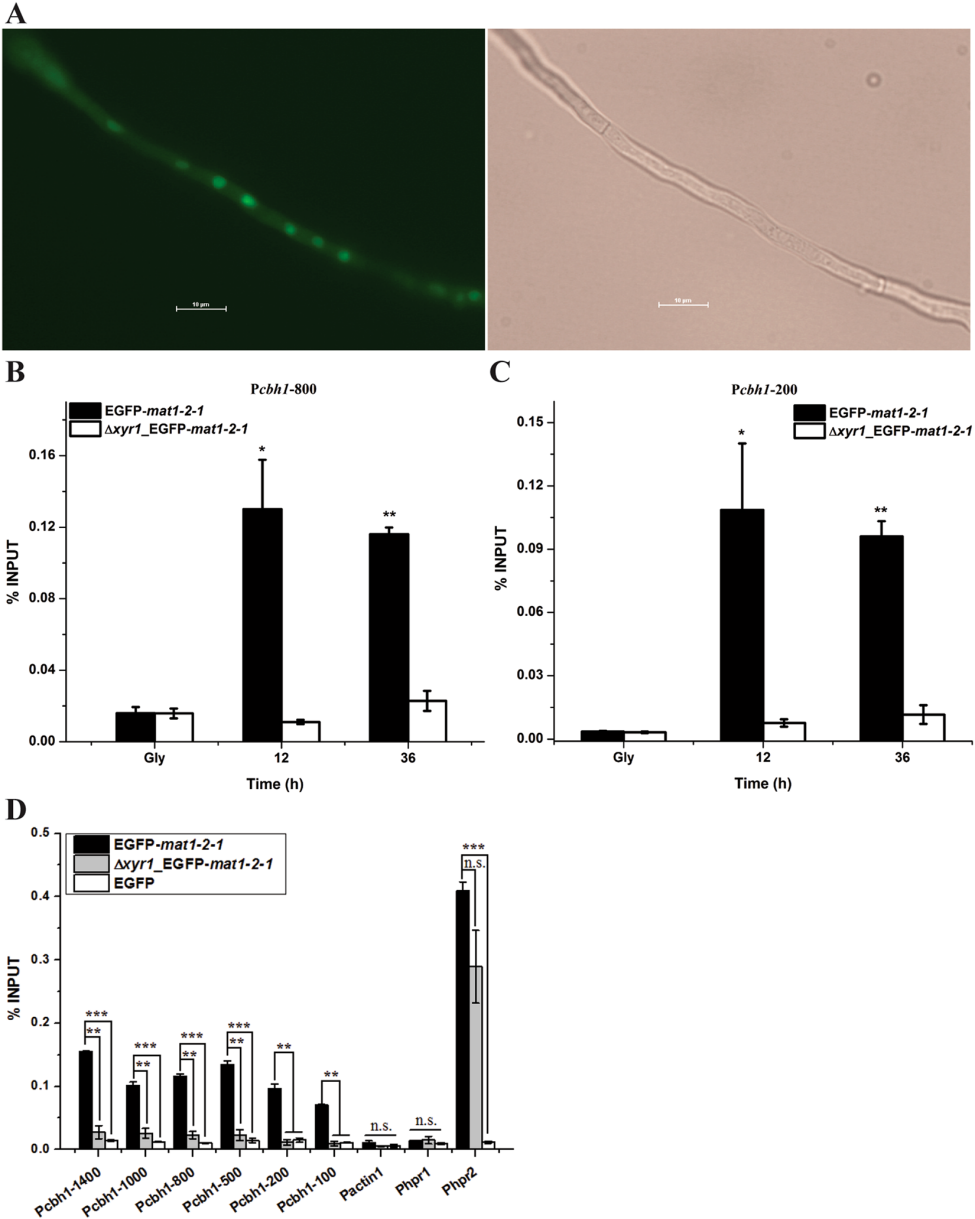
MAT1-2-1 recruitment to the *cbh1* promoter is dependent on Xyr1. (**A**) Expression and subcellular localization of EGFP-MAT1-2-1 in the Δ*xyr1* strain. (**B** and **C**) ChIP analyses of MAT1-2-1 recruitment to the −800 and −200 regions of the *cbh1* promoter in the presence or absence of Xyr1 after induction with Avicel for the indicated time periods. Gly denoted pre-cultures on glycerol. (**D**) ChIP analyses of MAT1-2-1 recruitment to different regions of the *cbh1* promoter under Avicel inducing conditions in the EGFP-MAT1-2-1, Δ*xyr1*_EGFP-MAT1-2-1 strains and a control strain expressing EGFP. Error bars indicated the SD from two independent biological replicates. The *actin*, *hpr1* and *hpr2* promoters were used as controls. Significant differences (T-test *P < 0.05, **P < 0.01, ***P < 0.001, n.s. not significant) were detected for the recruitment of EGFP-MAT1-2-1 to all the tested regions of the *cbh1* promoter between the EGFP-MAT1-2-1 and the Δ*xyr1*_EGFP-MAT1-2-1 strains. No significant recruitment was detected for MAT1-2-1 to the *actin* and *hpr1* promoters or for GFP alone to all the tested promoters.

To elucidate whether the MAT12-1 recruitment to the *cbh1* promoter is dependent on Xyr1, a recombinant strain expressing GFP-MAT1-2-1 but with *xyr1* deleted was constructed and the same ChIP experiments were repeated. As shown in Fig. 8, whereas the appropriate expression and nuclear localization of GFP-MAT1-2-1 was hardly affected by the absence of Xyr1 (Fig. 8A), its enrichment on all tested *cbh1* promoter regions was almost abolished in the absence of Xyr1 (Fig. 8B–D). On the contrary, MAT1-2-1 was still capable of being recruited to the *hpr2* promoter (Fig. 8D). The same ChIP assay with a control strain expressing EGFP alone revealed that there were no recruitment signal of EGFP to all the tested promoters (Fig. 8D). Taken together, these results indicated that MAT1-2-1 may be directly involved in regulating cellulase gene expression by being specifically recruited to their promoters, and that its recruitment is most likely mediated by its interaction with Xyr1.

## Discussion

The regulation of cellulase gene expression has been extensively investigated in *T. reesei*, and the process has been shown to be controlled by a suit of transcription factors^3,24–27^. Whereas Xyr1 is the central regulator and absolutely necessary for activating the expression of most cellulases and hemicellulases^3^, several lines of evidence strongly suggest that fine-tuning of (hemi)-cellulase expression in *T. reesei* is achieved by an as-yet-unknown robust regulatory network involving the combinatorial and synergistic actions of different repressors and activators bound at multiple sites of the promoter^27,28^. In this study, we identified the mating type locus protein MAT1-2-1 of *T. reesei* that interacts with Xyr1, binds to the *cbh1* promoter and modulates cellulase gene expression. As a conserved transcription factor (TF), MAT1-2-1 contains a high mobility group (HMG) domain that has no similarity to *S. cerevisiae* mating-type TFs, but is found in TFs of diverse lower or higher eukaryotes. Functional analyses in *Penicillium chrysogenum* provided evidence that MAT1-2-1 has functions beyond sexual development though the precise underlying mechanism is unclear^29^. In fact, HMG protein has been implicated in the regulation of transcription through interacting both with the basal transcription machinery and with individual TFs^30,31^. Once localized for binding, HMG proteins have been also reported to play an important role in maintaining chromatin structure and as a co-regulator of transcription through its strong DNA bending activity^32^. In our *in vitro* studies, we found that MAT1-2-1 directly interacted with the putative AD and MHR2 regions of Xyr1. Further ChIP assay revealed that MAT1-2-1 was recruited to the endogenous *cbh1* promoter in an induction and Xyr1-dependent manner. The exact reason for the almost evenly recruitment of MAT1-2-1 to all the tested *cbh1* promoter regions was not clear at present. One possible explanation is that, once MAT1-2-1 was recruited to some specific regions bound preferentially by Xyr1, it could somehow spread to the neighboring regions of the promoter. Nevertheless, the significant down-regulation of the induced cellulase gene expression in the absence of MAT1-2-1 under conditions wherein the endogenous *mat1-2-1* was otherwise up-regulated, strongly supports an important role of MAT1-2-1 as a physiological cofactor of Xyr1. The negligible effect of *mat1-2-1* deletion on the induced expression of cellulase genes in darkness is largely due to the fact that *mat1-2-1* was expressed at a much lower level compared to that in light.

**Figure 9:**
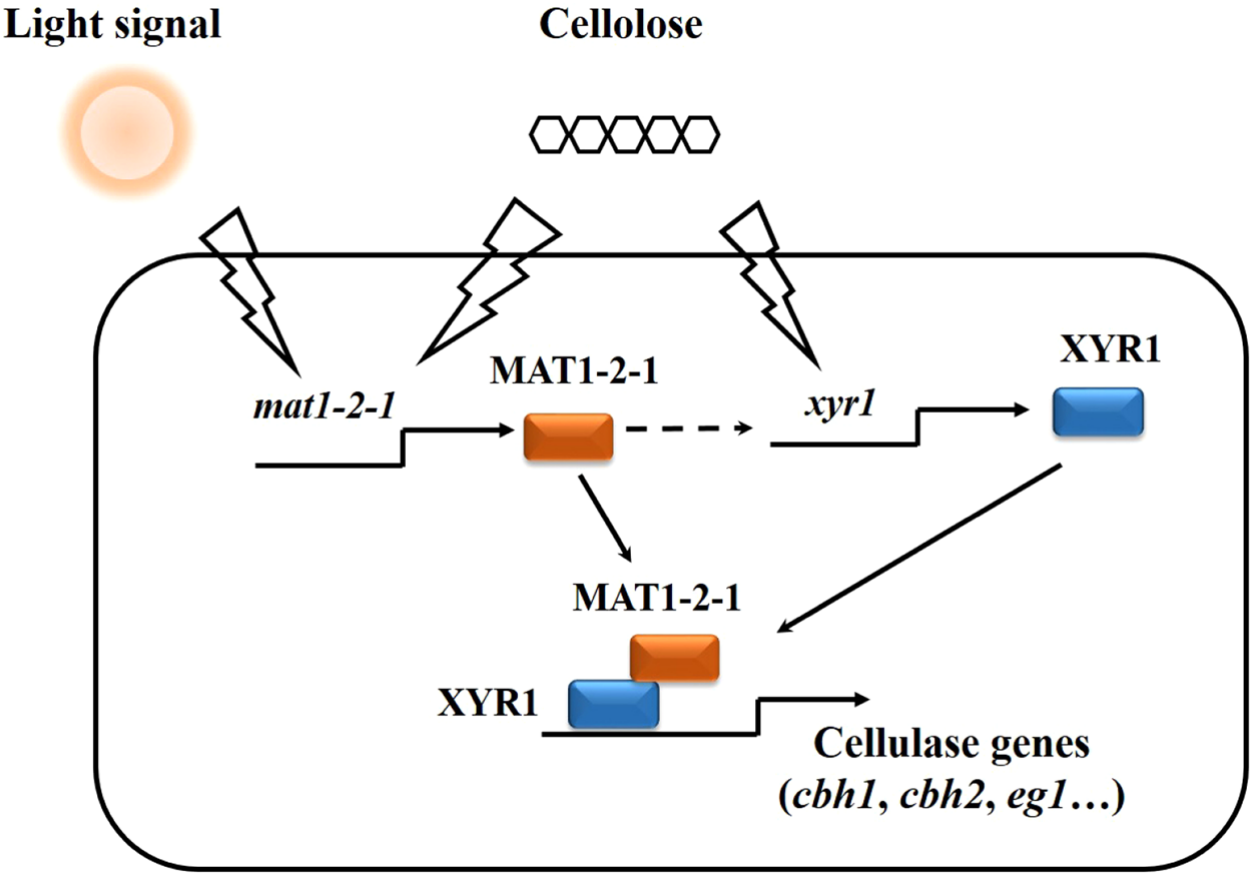
The putative regulatory effect of MAT1-2-1 on the cellulolytic response in *T. reesei*.

In addition to the type of carbon source, additional environmental conditions are known to affect *T. reesei* cellulase production. Among others, light is a crucial environmental factor for fungi wherein the light-induced changes in gene expression impact various physiological processes including growth, asexual and sexual reproduction, pigment formation, carbon metabolism, and circadian rhythms^33–35^. Interestingly, it has been found that the induction of cellulase gene transcription in *T. reesei* by cellulose is enhanced by light^7^. Although the effect of light on cellulase expression is likely not a direct effect, possibly an indirect one through general effects on metabolism, evidence exists that this light-dependent regulation is impacted by the PAS domain light-regulatory protein ENVOY (ENV1)^7,10^. Besides representing an important node in integrating signals from light and nutrients via heterotrimeric G-protein and cyclic AMP (cAMP) pathways, ENV1 has been also shown to be important for successful mating by balancing the levels of genes in light at least in part by exerting a negative effect on *MAT1-2-1* ^16^. In the absence of *env1*, a much dramatic up-regulation of *MAT1-2-1* was observed in daylight^16^. Although possibility that ENV1 participates in regulating cellulase gene expression in response to light via alternative signal effectors could not be excluded, the observation that MAT1-2-1 acts as a cofactor of Xyr1 promoted us to speculate that the abnormally higher up-regulation of MAT1-2-1 than that observed in WT could otherwise in part account for the retarded cellulase induction as displayed in the Δ*env1* mutant^7^ by otherwise interfering with Xyr1 function. Our data thus supports a regulatory model where MAT1-2-1 functions during the cellulolytic response in *T. reesei* (Fig. 9). Upon cellulose induction in light, both Xyr1 and an appropriate level of MAT1-2-1 are induced and then synergize in binding to cellulase gene promoters, thus leading to the efficient induction of cellulolytic genes. The mating-type-dependent effect on cellulase gene expression thus suggests that MAT1-2-1 may represent another regulatory node that integrates the light response with carbon source signaling to fine tune cellulase gene transcription.

## Materials and Methods

### Strains, medium, and cultivations

The uridine-auxotrophic strain *T. reesei* TU-6 (ATCC MYA-256) was used as a parental strain for targeted gene knock-out. A recombinant TU-6 strain with a retransformed *pyr4* gene termed TU6-RP, was used as control strain for all the phenotypic analysis experiments^24^. *T. reesei* strains were maintained on malt extract agar (Sigma-Aldrich, Madison, USA) supplemented with 10 mM uridine when necessary. For the transcription and cellulase production analyses, *T. reesei* strains were pre-grown in 1-liter Erlenmeyer flasks on a rotary shaker (200 rpm) at 30 °C in 250 ml Mandels-Andreotti medium with 1% glycerol (v/v) as the carbon source for 48 h. Mycelia were harvested by filtration and washed twice with Mandels-Andreotti medium with no carbon source. Equal amounts of mycelia were then transferred to a fresh medium containing 1% Avicel (w/v) (Sigma-Aldrich) or other carbon sources as indicated, and the incubation was continued for the indicated time periods.

The *Saccharomyces cerevisiae* strain Y2H Gold (MATa, trp1-901, leu2-3, 112, ura3-52, his3-200, gal4Δ, gal80Δ, LYS2::GAL1_UAS_–Gal1_TATA_–His3, GAL2_UAS_–Gal2_TATA_–Ade2 URA3::MEL1_UAS_–Mel1_TATA_ AUR1-C MEL1) (Clontech) was used as the host for the two-hybrid screen. Yeast cells were routinely cultivated at 30 °C in YPD medium (1% yeast extract, 2% peptone and 2% glucose). Synthetic complete (SC) medium lacking tryptophan and leucine with 100 ng/ml of Aureobasidin A (AbA, Clontech) was used for transformant selection. For plate growth assays, yeast cells with serial dilutions were spotted onto a selective quadruple dropout medium (QDO, SD/–Ade/–His/–Leu/–Trp) containing 100 ng/ml of AbA.

*Escherichia coli* DH5α cells were used for plasmid construction and amplification. *Escherichia coli* BL21 (DE3) cells were used as a host for expression of the recombinant proteins.

### cDNA library construction and yeast-based two-hybrid screening

Preparation of the cDNA library from mycelia of the strain QM9414 induced on Avicel was carried out as described previously by Cao Y *et* *al*.^24^.

For constructing the bait vector for yeast two-hybrid screening, the Xyr1 coding sequences either for its putative activation domain (AD, aa 767~940) or the middle homology domains (MHR1, aa 314~772 and MHR2, aa 314~632) were amplified from *T. reesei* cDNA with primers containing *Nde*I and *Bam*HI sites. The digested DNA fragments were ligated into pGBKT7 (Clontech) to obtain pGBKT7-Xyr1AD, pGBKT7-MHR1 and pGBKT7-MHR2, respectively.

The bait plasmid pGBKT7-Xyr1AD was transformed into the yeast Y2H Gold strain by a PEG-LiAC method to obtain the bait strain^36^. Ten micrograms of *T. reesei* cDNA library plasmid was then transformed into the bait strain and transformants were selected on SC plates lacking tryptophan and leucine but containing 100 ng ml^-1^ AbA. Growing colonies were picked and the harbored plasmids with cDNA insert were verified by DNA sequencing after retransformation.

### Plasmids and recombinant *T. reesei* strain construction

To delete *mat1-2-1* in TU-6, two DNA fragments corresponding to approximately 2-kb of *mat1-2-1* up- and downstream of the open reading frame (ORF) regions were amplified from *T. reesei* TU-6 genomic DNA and ligated into the pUC*pyr4* plasmid^37^ to yield the knock-out vector pUC*pyr4*-del*MAT1-2-1*, which was used to transform *T. reesei* TU-6 after linearization with *Eco*RI.

Transformation of *T. reesei* protoplast was carried out essentially as previously described^37^. Transformants were selected on minimal medium either for uridine prototroph or for resistance to hygromycin B (120 µg ml^-1^) after at least three rounds of single conidium purification.

To determine the subcellular localization of MAT1-2-1 and Xyr1, the *tcu1* gene (Tr_52315) promoter and the *cbh2* terminator were first amplified from QM9414 genomic DNA and inserted into the *Hin*dIII/*Sal*I and *Spe*I/*Sal*I sites in the pUC19-*hph* and pUC19-*pyr4* plasmids^37^ to obtain P*tcu1*-*hph* and P*tcu1*-*pyr4*, respectively. The EGFP and mCherry coding genes were then ligated into P*tcu1*-*hph* and P*tcu1*-*pyr4* after digestion with *Eco*RV and *Not*I to generate P*tcu1*-EGFP-*hph* and P*tcu1*-mCherry-*pyr4*, respectively. The *MAT1-2-1* and *Xyr1* coding sequences were finally amplified from *T. reesei* cDNA and inserted into the *Not*I and *Spe*I sites in the above two vectors to obtain P*tcu1*-EGFP-MAT1-2-1 and P*tcu1*-mCherry-Xyr1, respectively. These two intact vectors were co-transformed into TU-6 and P*tcu1*-EGFP-MAT1-2-1 was also transformed into the Δ*mat1-2-1* and Δ*xyr1* strains^3^.

### Recombinant protein production in *E. coli* and GST pull down

For the expression of Xyr1 MHR2, Xyr1 AD and MAT1-2-1 in *E. coli*, the DNA fragments coding for Xyr1 MHR2 and Xyr1 AD were amplified from TU-6 cDNA and ligated into the pGEX 4T-1 vector after digestion with *Eco*RI and *Not*I to obtain plasmids pGEX 4T-1-MHR2 and pGEX 4T-1-AD, respectively. The MAT1-2-1 coding sequence was amplified from TU-6 cDNA with primers harboring *Nde*I and *Eco*RI sites and ligated into pET22b (+) to obtain pET22b-MAT1-2-1.

The indicated expression constructs were transformed into CaCl_2_-treated competent *E. coli* BL21 (DE3) cells^38^. Protein purification was carried out essentially as described by Cao Y *et al*.^24^. All of the protein preparations were stored at −80 °C in the presence of 20% (v/v) glycerol.

For GST pull-down assay, the purified 6 × His MAT1-2-1 was incubated with glutathione sepharose bead-coupled GST, GST-Xyr1 AD, and GST-Xyr1 MHR2, respectively, with rotation at room temperature for half an hour before being washed three times with PBST buffer (137 mmol/L NaCl, 2.7 mmol/L KCl,10 mmol/L Na_2_HPO_4_,2 mmol/L KH_2_PO_4_, 0.5% Triton X-100, pH 7.4). The proteins in the flow through as well as those retained on the beads were resolved by SDS-PAGE and detected by Western blot with anti-His antibody (Santa Cruz, sc-8036).

### Fluorescence microscopy

The subcellular localization of EGFP-MAT1-2-1 and mCherry-Xyr1 was analyzed using fluorescence microscopy essentially as described previously^37^.

### Enzyme activity analysis

Extracellular cellobiohydrolase and β-glucosidase activities were determined by measuring the amount of released p-nitrophenol using p-nitrophenyl-D-cellobioside (*p*NPC; Sigma) and p-nitrophenyl-β-D- glucopyranoside (*p*NPG; Sigma) as the substrates, respectively. The enzymatic reactions were performed essentially as described previously^39^. One unit (U) of *p*NPCase or *p*NPGase activity corresponds to the conversion of 1 μmol substrate per minute under the test conditions.

### Quantitative RT-PCR (qRT-PCR)

To determine *mat1-2-1* transcription under different cultivation conditions, the parental strain TU-6 was pre-grown in Mandels-Andreotti (MA) medium with 1% glycerol (v/v) for 36 h and then transferred into a fresh batch of MA medium supplemented with 1% glucose (w/v), 1% lactose (w/v) or 1% Avicel (w/v) as the sole carbon source. The culture was continued under constant light (LL) or darkness (DD) condition for another 24 h. As for the transcription of cellulase genes, the Δ*mat1-2-1* and TU6-RP strains were pre-grown in MA medium with 1% glycerol (v/v) for 48 h and then transferred into MA medium supplemented with 1% Avicel (w/v) or 1% lactose (w/v) as the sole carbon source under constant light (LL) or darkness (DD).

RNA extraction and qRT-PCR analyses were performed as described previously^37^. Data analysis was performed using the relative quantitation/comparative CT (ΔΔCT) method and was normalized to an endogenous control (*rpl6e* ^40^). Transcript level from cultures on glycerol was used as the reference and set to 1. Three biological replicates were performed for each analysis. The results and the error bars are the mean of and SE (standard error) from these replicates, respectively. Statistical analysis was performed using the student’s *t*-test when determining whether significant differences exist between TU6-RP and the Δ*mat1-2-1* strain for the extracellular activities and the transcription of the *cbh1* and *egl1* genes. Similar statistical analysis was also performed for the recruitment signal of EGFP-MAT1-2-1 to each regions of the *cbh1* promoter and the control promoter between recombinant strains with or without Xyr1.

### Chromatin immunoprecipitation (ChIP)

To analyze the binding of indicated proteins to specific promoter regions, the recombinant strain expressing EGFP-MAT1-2-1 was cultivated in liquid MA medium containing 1% Avicel for 12 h and 36 h, respectively. ChIP assays were performed according to a previously described protocol^41,42^. Briefly, the mycelia were fixed in minimal medium containing 1% formaldehyde at 30 °C for 10 min with shaking and followed with 125 mM glycine shaking for an additional 5 min, which were then collected, ground in liquid N_2_ and broken in lysis buffer (50 mM HEPES pH 7.5, 150 mM NaCl, 1 mM EDTA, 0.5% Triton X-100, 0.1% sodium deoxycholate, 0.1% SDS, 1 mM PMSF (phenylmethanesulfonyl fluoride), 1 μg ml^-1^ leupeptin, and 1 μg ml^−1^ pepstatin A) with ceramic beads (0.5 mm). Chromatin DNA was further sonicated to obtain an average DNA fragment size of approximately 500 bp. Immunoprecipitation was then performed with anti-GFP (#ab290, Abcam) or anti-Xyr1 antibody^24^ with equal amounts of extract proteins (2 mg) at 4 °C for 5 h. Quantitative PCR was finally performed with the precipitated chromatin DNAs using a Bio-Rad IQ2 thermocycler (Bio-Rad) (Takara). Relative enrichment of the DNAs was calculated as a percentage of the input DNA. Primers used in quantitative RT-PCR and ChIP were listed in Supplementary Table S1.

## Electronic supplementary material

Supporting information
