# Supplementary material for: The mating type locus protein MAT1-2-1 of Trichoderma reesei interacts with Xyr1 and regulates cellulase gene expression in response to light
Source: Sci Rep. 2017 Dec 11;7:17346. doi: 10.1038/s41598-017-17439-2 (PMC5725425; doi:10.1038/s41598-017-17439-2)
Supplement: Supplementary file 1 — Supporting information [file 41598_2017_17439_MOESM1_ESM.pdf]

Supporting information

The mating type locus protein MAT1-2-1 of *Trichoderma reesei* interacts with Xyr1 and regulates cellulase gene expression in response to light

Fanglin Zheng, Yanli Cao, Lei Wang, Xinxing Lv, Xiangfeng Meng, Weixin Zhang, Guanjun Chen, Weifeng Liu*

No.27 Shanda South Road, State Key Laboratory of Microbial Technology, School of Life Science, Shandong University, Jinan 250100, Shandong, P. R. China;

Running title: MAT1-2-1 modulates cellulase gene expression

Keywords: MAT1-2-1; Xyr1; cellulase; light; *Trichoderma reesei*

Table S1 Primers for quantitative RT-PCR and ChIP used in this study.

Primer name	Sequences (5' to 3')
mat1-2-1 qF	ATCGTCACAATCTGGTCAAGGCT
mat1-2-1 qR	ATTGGTAGTCGGGATGCTTCTCA
cbh1 qF	CTTGGCAACGAGTTCTCTT
cbh1 qR	TGTTGGTGGGATACTTGCT
eg1 qF	CGGCTACAAAAGCTACTACG
eg1 qR	CTGGTACTTGCGGGTGAT
crt1 qF	GATTCGGCGTCTCCATTG
crt1 qR	CGAGAACCAGAGAGTGTTG
xyr1 qF	GGTTGGACGACTGGATCT
xyr1 qR	GGTTGTCCTCCATGATGTAG
rpl6e qF	CGTCATCGCCACCTCCTACAA
rpl6e qR	GCTGAAGGTGCTGGCGAGGTA
Pcbh1-800 qF	GGCAAGGGAAACCACCGAT
Pcbh1-800 qR	TGGACTGAGTGAAGAAACAAACG
Pcbh1-200 qF	CCTTCGGCCTTTGGGTGTA
Pcbh1-200 qR	TCTTTATCGGCTATTGTTCTTGG
Pcbh1-100 qF	TAGCCAAGAACAATAGCCGATAA
Pcbh1-100 qR	TTTCTGTGCCTCAAAAGATGGT
Pcbh1-500 qF	GGCAGTGATGGAAGACAGTGAAA
Pcbh1-500 qR	TCGTCGTATCGGCAGACAAACCT
Pcbh1-1000 qF	CCTCTTCTCAACCTTTGGCGTTTC
Pcbh1-1000 qR	TCTACGAGCAGAGTTCGGATAACA
Pcbh1-1400 qF	CGAACCCGGAGAATCGAGATGTGC
Pcbh1-1400 qR	GCTACTGCGACGGAACGCTTTGCT
Phpp1 qF	GACGAGGACGACGAGGGCAAGA
Phpp1 qR	GCGCCAAAGTGAGCATTAAGGTAGG
Ppgg1 qF	GCTTTGATCCGCGTGTATCTC
Ppgg1 qR	CGTATTGTGCCATCAGCGTCTT
Phpr1 qF	AAACCTGGTCGTGGAGATGCC
Phpr1 qR	AATAAGAAGCAGGACTCAGAACTCG
Phpr2 qF	GCGACCTGCATTTAATCCCTT
Phpr2 qR	GGAATGGTAGGCTGAATCTGG

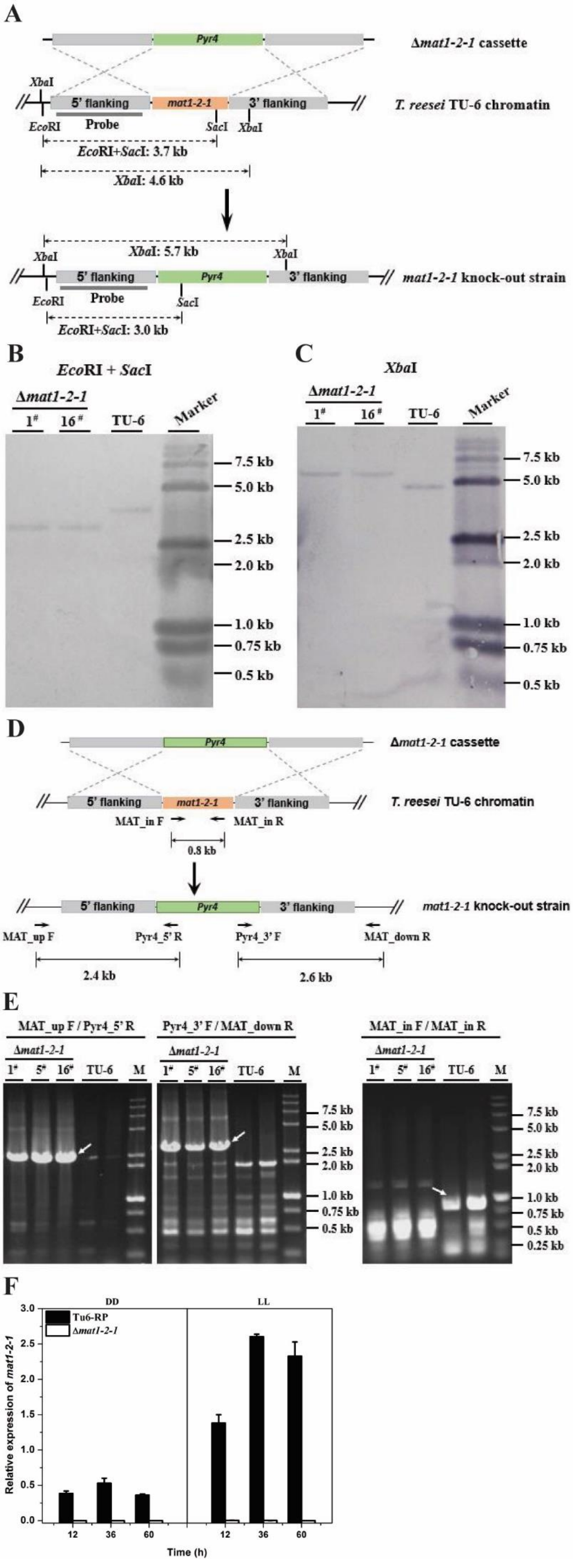
A
Pyr4
Δmat1-2-1 cassette
XbaI
5' flanking
mat1-2-1
3' flanking
T. reesei TU-6 chromatin
EcoRI
Probe
SacI
XbaI
EcoRI+SacI: 3.7 kb
XbaI: 4.6 kb
XbaI: 5.7 kb
XbaI
XbaI
5' flanking
Pyr4
3' flanking
mat1-2-1 knock-out strain
EcoRI
Probe
SacI
EcoRI+SacI: 3.0 kb
B
EcoRI + SacI
Δmat1-2-1
1#
16#
TU-6
Marker
7.5 kb
5.0 kb
2.5 kb
2.0 kb
1.0 kb
0.75 kb
0.5 kb
C
XbaI
Δmat1-2-1
1#
16#
TU-6
Marker
7.5 kb
5.0 kb
2.5 kb
2.0 kb
1.0 kb
0.75 kb
0.5 kb
D
Pyr4
Δmat1-2-1 cassette
5' flanking
mat1-2-1
3' flanking
T. reesei TU-6 chromatin
MAT_in F
MAT_in R
0.8 kb
5' flanking
Pyr4
3' flanking
mat1-2-1 knock-out strain
MAT_up F
Pyr4_5' R
Pyr4_3' F
MAT_down R
2.4 kb
2.6 kb
E
MAT_up F / Pyr4_5' R
Δmat1-2-1
1#
5#
16#
TU-6
M
Pyr4_3' F / MAT_down R
Δmat1-2-1
1#
5#
16#
TU-6
M
7.5 kb
5.0 kb
2.5 kb
2.0 kb
1.0 kb
0.75 kb
0.5 kb
MAT_in F / MAT_in R
Δmat1-2-1
1#
5#
16#
TU-6
M
7.5 kb
5.0 kb
2.5 kb
2.0 kb
1.0 kb
0.75 kb
0.5 kb
0.25 kb
F
DD
LL
Tu6-RP
Δmat1-2-1
Relative expression of mat1-2-1
3.0
2.5
2.0
1.5
1.0
0.5
0.0
12
36
60
12
36
60
Time (h)

Fig. S1 Verifying the Δ*mat1-2-1* transformants by Southern blot (A-C), genomic PCR (D and E) and RT-qPCR (F). DD, constant darkness; LL, constant light.

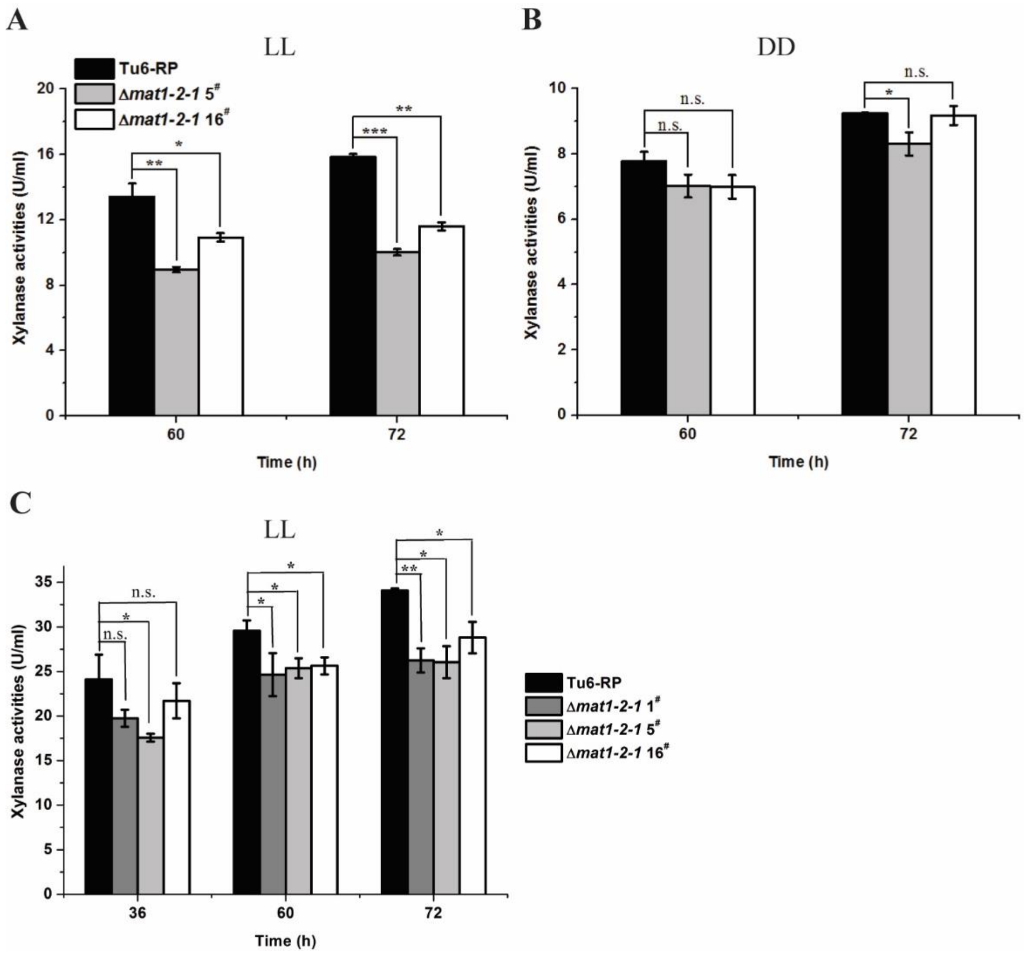

Fig. S2 Deletion of *mat1-2-1* resulted in reduced xylanase activities on cellulose or xylan in light. Xylan hydrolytic activities of the extracellular supernatant of two and three independent Δ*mat1-2-1* transformants and TU6-RP strains induced on cellulose (A-B) or on xylan (C)， respetively,

in light or darkness. (T-test, *P<0.05, **P<0.01, ***P<0.001, n.s. not signiicant)
